# Four Operations for Appendicitis: Three Recoveries; One Death from a Very Small Concealed Abscess

**Published:** 1891-12

**Authors:** W. W. Keen

**Affiliations:** Professor of Principles of Surgery, Jefferson Medical College


SECOND PAPER.

### FOUR OPERATIONS FOR APPENDICITIS: THREE RECOVERIES; ONE DEATH FROM A VERY SMALL CONCEALED ABSCESS.

By W. W. KEEN, M. D.,

Professor of Principles of Surgery, Jefferson Medical College.

CASE I. *Recurrent appendicitis; operation after the fifth attack (with perforation and general peritonitis), by median and lateral incisions; recovery.* Miss B., aged thirty years, a slender, frail woman. A year ago she developed a moderate lateral curvature of the spine through muscular weakness. Her father died of cancer of the bowels; her mother is living, and is even more delicate than herself.

About fifteen years ago she had her first attack of perityphlitis. A few years later a second occurred, and about six years ago a third, which was the first in which I saw her. The attack was not severe, no suppuration followed, and after its subsidence she seemed as well as usual.

On May 31, 1890, she was suddenly taken with severe paroxysmal pain in the lower part of the abdomen, accompanied by vomiting. The attack was attributed to the eating of some strawberries, and when the bowels were subsequently moved by small doses of calomel, a quantity of strawberry seeds was passed. The pain was relieved by morphia. There was slight general tenderness, not limited to the right iliac fossa. The temperature only rose to 101°. The attack gradually passed away, and in two weeks she was able to return home. For the account of this attack I am indebted to Dr. W. H. Morrison, who attended her. The symptoms rather pointed in his mind to an ordinary intestinal colic from the fruit, though, as there had been prior attacks of perityphlitis, the right iliac fossa was watched with some care; but no special dulness or tenderness existed there, nor was there any induration. There had been no chill.

The summer of 1890 was passed in comparatively good health. As soon as I returned from my summer holiday, I was asked to see her by my assistant, Dr. W. J. Taylor. He had diagnosticated not only a renewed attack, but also a probable perforation of the appendix, on the day of my return. She had been constipated for several days, and a slight movement of the bowels on September 30, 1890, due to divided doses of Epsom salt given the previous evening, was followed by symptoms of peritonitis over the entire lower abdomen, the tenderness in the left iliac fossa being possibly even more marked than in the right. The induration was only moderate. Exploration by the rectum revealed general tenderness of the pelvic viscera, with diffused induration, but no fluctuation. A vaginal examination could not be made. It was clear that perforation had taken place, and that immediate operation was needful.

When I first saw her on the evening of October 1st, the symptoms, while clear, were not very urgent, and Dr. Taylor and I felt it safe to postpone the operation until the next day, and so avail ourselves of daylight. The peritonitis was clearly local in the pelvis and lower belly; it did not involve the entire peritoneum, and the depression and shock were not so great as to require instant interference.

Operation, October 1, 1890. The hair was shaved and the field of operation thoroughly disinfected. In view of the involvement of both iliac fossa, I deemed it best to make an incision in the middle line. As soon as the abdominal wall was penetrated, pus began to exude very abundantly, and I estimated that over a pint escaped. The omentum was glued to the belly wall, and the pelvic viscera, including the intestines, were all glued together by adhesions, except where they were separated from each other by pus.

The appendix was at once sought for. It was firmly bound in place, as thick as a good-sized thumb, and so turgid that it was erect. It could not be brought to or even near the opening, and accordingly another oblique incision was made in the right iliac fossa, through which it was approached very readily. As soon as seen it was discovered that there was a small opening at its tip, through which the intestinal contents were escaping. With some little care it was freed from its adhesions, tied one-fourth of an inch from the cecum, and removed. The stump was then disinfected and invaginated into the bowel, the peritoneal coating of which was secured by four Lembert stitches. The entire abdominal cavity was then thoroughly flushed with hot water. Two drainage-tubes of glass were inserted, one in each incision, and the wounds closed. I sought to carry the drainage-tube in the middle line into Douglas's cul-de-sac, butthis could not be found, as it was obliterated by adhesions. The drainage-tubes were emptied by a long-nozzled syringe whenever full.

*December* 17, 1890. In twenty-four hours after the operation, without laxatives, her bowels were moved twelve times, and for a week afterward from two to four or six times a day, in small quantities, semisolid. The bladder was emptied most of the time by catheter. Her temperature, which two days before the operation had reached 103°, fell immediately after the operation to 101.6°, and four days after the operation had reached the normal. By the end of a week it had gradually climbed again to 102°, and on that day she had a severe "sinking spell," with much pain, cold perspiration, and excessively weak pulse. This was met by prompt administration of stimulants and digitalis, and in forty-eight hours her temperature had fallen about two degrees. Meantime her bowels coutinued to trouble her very greatly, with pain and frequent movements. Examination by the rectum showed the pelvic viscera to be matted together in a hard mass, which pressed upon the rectum and gave great annoyance. Until the eighteenth day after the operation her temperature fluctuated very markedly from normal or a little above to 101° to 102°, but on the nineteenth day, coincidently with the improvement in her bowels, her temperature sank to normal and remained such during the remainder of her convalescence. In fact, most of the time it was half a degree below normal.

Meantime the median wound gaped open to the extent of over an inch in consequence of the sloughing out of the stitches, but by the time it had gaped open a layer of granulation had sprung up on the omentum, which lay at its bottom, and without any interference other than the daily redressing,—sometimes several times a day, when the discharge was considerable,—it slowly healed, and by the end of five weeks was entirely closed by a firm cicatrix. The drainage-tube was removed at the end of ten days, when there was no further discharge through it. The lateral wound healed without incident at the end of ten days.

After the lapse of three weeks from the operation her progress was slow, but steady. A month after the operation she first sat up out of bed.

*September*, 1891. There is a slight tendency to a ventral hernia at each incision, for which she uses a binder. Her menstruation is regular and not especially painful. Examination of the pelvic viscera by the rectum shows them to be mobile and free from adhesions.

CASE II. *Perforative appendicitis of a week's duration; temperature of only 99° in spite of a large abscess; operation; recovery.*—H. T., male, aged forty-two years; admitted to the Jefferson College Hospital, February 11, 1891, at the request of Dr. C. M. Ellis, of Elkton, Md. Family history negative. He was taken ill on February 4th with pain all over the belly. This was not located in the right iliac fossa until two days later. His fever had run up to 102° and 103°. There was tumefaction in the right iliac fossa, but no edema. There was resistance to touch, parallel with Poupart's ligament, and filling up half of the space between Poupart's ligament and the umbilicus. The point of greatest tenderness was one inch below McBurney's point. On the evening of his admission his temperature was only 100°, and was no higher the next morning. On the evening of the second day, however, the temperature rose to 101.2°, falling the next morning to 99°. Finding that the vesperal rise of temperature continued, and that a week had elapsed, I determined at once to operate. This was done in the public clinic.

An oblique lateral incision was made parallel to Poupart's ligament, which immediately liberated a large quantity of very foul smelling pus. The appendix was found to be swollen to about the size of the thumb, with a distinct perforation of the diameter of a knitting-needle at its extremity. The appendix was tied and cut off. A second smaller abscess cavity was found at a deeper level than the first, the pus from which was much more fetid than that from the first. The cavity was then thoroughly washed out with a sterilized salt solution, a drainage tube was inserted, and the centre of the incision united by a few stitches. Practically, these might well have been omitted, for at the end of three weeks, in order to secure free drainage of the wound, it was necessary to lay open this adherent skin. He went home with a small, almost healed ulcer, nine weeks after the operation. The highest temperature was 102.2°, on the sixth day.

CASE III. *Appendicitis from a fecal concretion; pus in the general peritoneal cavity; operation ninety to ninety-six hours after inception of the disease; recovery.*—J. S., a French lad, aged nineteen years. Admitted to the Jefferson College Hospital, February 28, 1891. Four days before this he was in perfect health, and at his work as a waiter in a restaurant. He had never had a similar attack. On that day he was seized with cramps all over the belly. By the next day the pain had become fixed in the right iliac fossa, and I was called to see him on the morning of the fourth day by Dr. J. C. Wilson, who had been called in on the previous evening. When I entered the room the tears were rolling down his cheeks, and he was groaning and writhing with pain. The bowels had been opened on the day of the attack, but not since. On the evening of the third day the temperature was 103°, and on the morning of the fourth day 101°. There was tumefaction parallel to Poupart's ligament, about three fingers' breadth in width, and the anterior wall of the belly and right side was tense, elastic and resistant to the touch. It was extremely tender, and he indicated by one finger the tenderest spot at McBurney's point and one inch below it. The right leg was flexed to relax the belly wall. There was dulness on percussion, and a rectal examination showed considerable induration and obscure fluctuation. There was no edema. There had been no vomiting.

As quickly as possible arrangements were made to operate, and the operation was done before the class two hours after I first saw him with Dr. Wilson, as nearly as could be determined, between ninety and ninety-six hours after the attack. The incision was parallel to Poupart's ligament. Although there was no external edema, as soon as I cut through the aponeurosis of the external oblique, there was marked edema of the tissues beneath this aponeurosis. At a greater depth a quantity of extremely fetid, very thin pus gushed out, and on washing out the cavity with a salt solution, I found an evidently gangrenous mass, with a knobbed free end, which looked like the appendix bound down by adhesions. A piece of the omentum was attached to it, and the appendix was distended, but clearly not perforated. It was ligated and cut off. Upon opening the amputated portion, I found a fecal concretion as large as a bean. So far as gentle manipulation could determine, there seemed to be no adhesion of the bowels to each other, and apparently the pus was contained in the general peritoneal cavity. This was well washed out with hot water and closed, and a drainage-tube was inserted. The patient made a rapid recovery without any serious complications whatever, improving from the very moment of the operation. He went home in three weeks entirely well.

Case IV. *Perforative appendicitis; pain below the ribs; laparatomy; death; concealed small abscess behind the colon.*—Mrs. F., American, aged thirty years, was first seen at 11.30 P. M., June 27, 1891, with Dr. Seitz. She had married at fifteen, and has had four children, – the last, three years ago. She has been perfectly regular, the last sickness coming on a week too early, ten days ago. A week ago she was suddenly seized with violent pain just below the right border of the ribs. A day or two later one of her children struck her accidentally over the same spot, producing intense pain. Five days ago she was seized with an aggravation of the pains, and was in such a condition of collapse that Dr. Seitz feared she would die. Her temperature was below 97°. Active stimulation soon relieved this, but the pain continued almost as severe as before. Another attack of collapse today, with cold extremities up to the knees and elbows, induced Dr. Seitz to call me in consultation. I found a slender, delicate-looking woman, with the right leg drawn up and the right side of the abdomen excessively tender, with the muscular wall of the belly very tense. The slightest touch on the entire right side of the abdomen produced the most severe pain. On the left side moderate pressure was pretty well borne. The pain was most severe just below the border of the liver, diminishing gradually toward the right iliac fossa. The uterus and ovaries, by vaginal touch, were free from pain and swelling.

At the consultation it was decided to give her hypodermatics of morphia, with brandy and milk, and in the morning, if she was not better, to do an exploratory laparatomy.

*June* 28, 11 A. M. The pain continued as bad as before, with the extremities cold and pulse irregular—92 to the minute – respiration, 24; temperature. 97.4°. An exploratory laparatomy was done, the incision being at the border of the right rectus. The diagnosis had been that of appendicitis, or some indeterminate trouble with the liver or gall-bladder. The kidney did not seem to be tender. On opening the abdomen the lower border of the liver was seen, and was evidently somewhat reddened and fleshy-looking. This was bound to the colon by recent adhesions, and the peritoneum of the corresponding belly-wall was deeply injected. The gall-bladder was normal, and there was no evidence of trouble behind the colon (see "Remarks" below) or with the kidney. No abscess or other cause for the inflammation could be detected. The right iliac region and caput coli showed no disease, but the appendix was not found. There was a considerable accumulation of serum in the right flank. The intestines were normal, also the uterus and the right ovary. In the left ovary was a small cyst. The abdomen was well flushed with warm water, and reluctantly closed after inserting a drainage-tube in the affected area. I felt assured that I had not discovered the reason for her dangerous illness.

4 P. M. She was much more comfortable than before the operation, and her extremities, though not warm, were much less cold.

*29th.* She passed a poor night, with constant bilious vomiting. Temperature, 97.4° ; extremities again cold. We ordered one-quarter of a grain of cocaine every hour, and a full enema with glycerin, followed, if need be, by an enema of two drachms of sulphate magnesia every two hours.

6 P. M. Temperature, 98.2°; pulse, 92; respiration, 24. Has had four large stools, and feels much more comfortable. The belly is not nearly so tender. The vomiting ceased with the first dose of cocaine, and she feels hungry. A moderate amount of bloody serum had escaped by the tube, which was now removed. A considerable amount of apparently purulent, leucorrheal discharge had occurred during the day.

*July* 2. From the time of the last note she gradually sank, with symptoms of collapse, subnormal temperature, and constant vomiting, until she died, at 9 P. M., on the 30th.

The post-mortem, thirteen hours after death, disclosed the fact that her death was caused by a perforative appendicitis. The appendix was three inches long, and lay directly behind the cecum and colon, being agglutinated to them, with no peritoneal covering, but lying between the two layers of the meso-colon. Its tip was perforated. Less than two drachms of pus, mixed with a small amount of fecal matter, was found in the abscess. The wound itself and the peritoneal cavity were entirely aseptic.

### REMARKS.

I record this case especially as a lesson in diagnosis and a warning in treatment. When first called to see it, the history, the collapse, the rigidity of the right side of the belly, and the flexure of the right leg, all betokened an appendicitis. And yet the right iliac fossa was free from tenderness, free from tumor, free from edema, free from pain. There was a slight pain and tenderness all over the right half of the belly, but the most painful spot was far away from McBurney's point, and was just under the border of the liver and about an inch inside the line of the anterior superior spine. The abdomen at this point, over an area of 2.5 to 3 inches, was so exquisitely tender, that no satisfactory examination could be made. Although appendicitis was in my mind as a first thought, the position of the tenderness suggested probable rupture of the gall-bladder from gall-stones, or a renal calculus, as the probable cause. When the abdomen was opened, the localized patch of peritonitis was external to the attachments of the mesocolon, and showed no indication of any trouble back of the colon as its possible cause. In spite of this, however, I examined three several times, with the most minute care, the entire region of the colon, from the cecum to the hepatic flexure; first on its outer side, then on its inner side, and then by bi-manual examination from side to side, and by palpation from before backward, but could detect no hardness or other evidence of any abscess.

That no larger an amount of pus should have formed after an illness lasting eight days is very unusual, and while I deeply regret not having discovered the abscess, I cannot but console myself with the thought that it was not for the want of a careful and thorough search, but by reason of the unusual conditions and the small size of the abscess. Whether in the absence of all physical signs of such an abscess it would have been my duty to dissect up the colon in order to examine the recto-colic tissues and appendix, or to have torn through the outer layer of the mesocolon, is a question I have much debated. Viewing now the facts, I greatly regret not having done so, and I report the case especially as a guide and warning to other surgeons who may meet with similar cases.

